# Full-length transcriptome assembly of *andrias davidianus* (amphibia: caudata) skin via hybrid sequencing

**DOI:** 10.1042/BSR20210511

**Published:** 2021-08-02

**Authors:** Yu Bai, Yonglu Meng, Jianlin Luo, Hui Wang, Guoyong Li, Can Li

**Affiliations:** 1 College of Mathematics and Information Science, Guiyang University, Guiyang 550005, China; 2 Guizhou Provincial Key Laboratory for Rare Animal and Economic Insects of the Mountainous Region, Guiyang University, Guiyang 550005, China; 3 Collaborative Innovation Center of Sustainable Utilization of Giant Salamander in Guizhou Province, Guiyang University, Guiyang 550005, China

**Keywords:** Andrias davidianus, Chinese giant salamander, hybrid sequencing, Illumina, PacBio, skin transcriptome

## Abstract

The Chinese giant salamander, *Andrias davidianus*, is the largest amphibian species in the world; it is thus an economically and ecologically important species. The skin of *A. davidianus* exhibits complex adaptive structural and functional adaptations to facilitate survival in aquatic and terrestrial ecosystems. Here, we report the first full-length amphibian transcriptome from the dorsal skin of *A. davidianus*, which was assembled using hybrid sequencing and the PacBio and Illumina platforms. A total of 153,038 transcripts were hybrid assembled (mean length of 2039 bp and N50 of 2172 bp), and 133,794 were annotated in at least one database (nr, Swiss-Prot, KEGG, KOGs, GO, and nt). A total of 58,732, 68,742, and 115,876 transcripts were classified into 24 KOG categories, 1903 GO term categories, and 46 KEGG pathways (level 2), respectively. A total of 207,627 protein-coding regions, 785 transcription factors, 27,237 potential long non-coding RNAs, and 8299 simple sequence repeats were also identified. The hybrid-assembled transcriptome recovered more full-length transcripts, had a higher N50 contig length, and a higher annotation rate of unique genes compared with that assembled in previous studies using next-generation sequencing. The high-quality full-length reference gene set generated in this study will help elucidate the genetic characteristics of *A. davidianus* skin and aid the identification of functional skin proteins.

## Introduction

Amphibian skin has evolved diverse functions to facilitate adaptation to the external environment [1]. The granular glands of amphibian skin produce four types of biologically active compounds [2–4]: (1) biogenic amines, (2) bufadienolides (bufogenins), (3) alkaloids and steroids, and (4) peptides and proteins [2]. Functional research of these substances aids the protection of giant salamanders and the development of medicinal materials, such as antimicrobial peptides and collagen. Upon contact with an external stimulus, the skin granular glands of the Chinese giant salamander (*Andrias davidianus*) secrete transparent viscous substances. However, the structure of these secretions remains unclear, largely because these secretions cannot be dissolved in conventional solvents such as acetic acid, ethanol, and acetone. Transcriptome sequencing of the skin can be used to determine the protein sequences of skin-secreted proteins, but this requires obtaining high-quality skin transcripts of *A. davidianus*.

To date, several transcriptomes of *A. davidianus* have been sequenced and published. A total of 147 transcripts have been shown to be involved in the immune responses and inflammatory reactions based on transcriptomes of spleen and skin tissue [5]. A transcriptome analysis has been conducted on the spleen tissue of *A. davidianus* during the host response to iridovirus infection [6]. A differential transcriptome analysis has been conducted on the spleen, heart, and liver tissues of the giant salamander in response to *Aeromonas hydrophila* [7]. A transcriptome analysis was performed on the spleen tissues of *A. davidianus* [8]. RNA sequencing (RNA-seq) data were obtained from a pool of *A. davidianus* tissues including spleen, liver, muscle, kidney, skin, testis, gut, and heart [9,10]. The transcriptome has been sequenced and assembled from multiple tissues (abdominal skin, dorsal skin, lateral skin, lung, heart, kidney, liver, pancreas, small intestine, spleen, stomach, brain, spinal cord, cartilage, eye, fingertip, long bone, maxillary, skull, muscle, ovary, fat, tail fat, and blood) [11]. A comparative transcriptomic analysis revealed the genetic basis underlying the immune function of the skin in three amphibians [12]. A high-coverage reference transcriptome was generated from gill, lung, and skin tissues of metamorphosing juvenile *A. davidianus* [13]. The transcriptomic responses of species have been shown to vary in response to ranavirus infection [14]. A comparative transcriptomic analysis of the ovaries and testes revealed genes that facilitate adaptation to the environment as well as important sex-biased genes in *A. davidianus* [15,16]. An RNA-seq analysis from different parts of the skin has provided insight into the molecular adaptations of *A. davidianus* [17].

*Andrias davidianus* not only is the largest amphibian species in the world but also has a large and complex genome (up to 50 Gbp; 2*n* = 60) [18,19], making *de novo* genome assembly for this species impractical using current sequencing technologies. Transcriptome sequencing is needed to study the transcriptional regulation of *A. davidianus*. However, errors often occur in the assembly of short reads produced by next-generation sequencing (NGS), and these errors cannot be corrected by the genome. Despite the remarkable developments in sequencing methodologies, there is a need to develop novel tools that facilitate comprehensive analyses of large quantities of sequence data and that can generate high-quality full-length reads from long-read sequencing data for analyses of short sequence reads [20,21].

Here, we used single-molecule real-time long-read technology to sequence the transcriptome of *A. davidianus* (which lacks a complete genome) using the PacBio Sequel platform. The aims of the present study were to produce an accurate full-length skin transcriptome of *A. davidianus*, which was used as a reference dataset along with the proteome and metabolome to explore the functional protein and gene families on the skin.

## Materials and methods

### Animal materials

A healthy female *A. davidianus* (body length, 70 cm; weight, approximately 3 kg; age, 4 years) was obtained from a farm in Guiding County, Guizhou Province, China. In the Collaborative Innovation Center of Sustainable Utilization of Giant Salamander, *A. davidianus* was stunned by a 100-V electric shock on the head; its body was then placed ventral side up, and its throat was cut with a knife. Dorsal skin tissues were immediately dissected from the giant salamanders and washed in sterile PBS. Animal tissue samples for RNA extraction were snap-frozen in liquid nitrogen and stored at −80°C until analyses.

### RNA preparation

Total RNA was prepared by grinding tissue in TRIzol reagent (Invitrogen 15596026) on dry ice and processed per the manufacturer’s protocol. Precipitated RNA was stored at –20°C until analysis.

### PacBio library preparation, sequencing, and data processing

The Iso-Seq library was prepared per the Isoform Sequencing protocol (Iso-Seq) using a Clontech SMARTer PCR cDNA Synthesis Kit (Catalog No. 634925). We constructed two size-fractionated libraries (0.5–4 kb and >4 kb) using the BluePippin Size Selection System and the protocol described by Pacific Biosciences (PN 100-092-800-03). The library preparations were sequenced on the PacBio Sequel platform (Pacific Biosciences, Inc., Menlo Park, CA, U.S.A.). Sequence data were processed using SMRT link 5.0 software (https://github.com/PacificBiosciences/SMRT-Link). Circular consensus sequences (CCSs) were generated from subread BAM files with the following parameters: min_length 200, max_drop_fraction 0.8, no_polish TRUE, min_zscore -999, min_passes 1, min_predicted_accuracy 0.8, and max_length 18000. CCS.BAM files were output and then classified into full-length and non-full-length reads using pbclassify.py (https://github.com/PacificBiosciences/pbtranscript/blob/master/pbtranscript/tasks/classify.py) with the following parameters: ignorepolyA false and minSeqLength 200. Non-full-length and full-length FASTA files were then fed into the cluster step for isoform-level clustering, followed by final polishing, using the following parameters: hq_quiver_min_accuracy 0.99, bin_by_primer false, bin_size_kb 1, qv_trim_5p 100, and qv_trim_3p 30. We combined the two size-fractionated libraries using Cd-Hit software with a similarity threshold of 99%.

### Illumina library preparation, sequencing, and data processing

Three-microgram RNA samples were used for RNA sample preparation. A sequencing library was generated using NEBNext Ultra™ RNA Library Prep Kit for Illumina (NEB, U.S.A.) per the manufacturer’s recommendations. Briefly, mRNA was purified from total RNA using poly-T oligo-attached magnetic beads. Fragmentation was carried out using divalent cations under elevated temperature in NEBNext First Strand Synthesis Reaction Buffer (5×). First-strand cDNA was synthesized using random hexamer primers and M-MuLV Reverse Transcriptase (RNase H). Second-strand cDNA synthesis was subsequently performed using DNA Polymerase I and RNase H. Remaining overhangs were converted into blunt ends via exonuclease/polymerase activities. After adenylation of the 3′ ends of DNA fragments, NEBNext Adaptors with hairpin loop structure were ligated in preparation for hybridization. To select cDNA fragments 350 bp in length, the library fragments were purified with an AMPure XP system (Beckman Coulter, Beverly, U.S.A.). Next, 3 µl of USER Enzyme (NEB, U.S.A.) was used with size-selected, adaptor-ligated cDNA at 37°C for 15 min, followed by 5 min at 95°C before PCR. PCR was performed with Phusion High-Fidelity DNA polymerase, Universal PCR primers, and Index (X) Primer. PCR products were purified (AMPure XP system), and library quality was assessed on an Agilent Bioanalyzer 2100 system. The clustering of the index-coded samples was performed on a cBot Cluster Generation System using TruSeq PE Cluster Kit v3-cBot-HS (Illumina, Inc., San Diego, CA, U.S.A.) per the manufacturer’s instructions. After cluster generation, the library preparations were sequenced on the Illumina NovaSeq 6000 platform (Illumina, Inc., San Diego, CA, U.S.A.), and 150-bp paired-end reads were generated. Raw data (raw reads) in FASTQ format were first processed through in-house perl scripts. Clean data (clean reads) were obtained by removing reads containing adapters, reads containing poly-N, and low-quality reads from raw data. Q20, Q30, and the GC content of the clean data were calculated. All downstream analyses were based on clean data with high quality. The resulting high-quality cleaned reads were assembled *de novo* into contigs using Trinity v2.4.0 [22] with “min_kmer_cov” set to 2. Corset v1.05 [23] was used to hierarchically cluster transcripts and obtain unigenes by comparing the number of reads and the expression patterns of transcripts. Short reads from the Illumina platform were aligned to transcripts using Bowtie2 version 2.2.5 [24] with a mismatch of 0.

### Correction, functional annotation, and analysis of transcripts

Polished consensus sequences from the PacBio platform were corrected using short reads from the Illumina platform by LoRDEC software [25] with 23 k-mers and a solidity abundance threshold of 3 for k-mers. To obtain annotation information, corrected transcripts were screened against the following databases using BLAST software version 2.2.28 (http://blast.ncbi.nlm.nih.gov/Blast.cgi) [26,27]: NCBI non-redundant protein sequences (nr); NCBI non-redundant nucleotide sequences (nt); KOGs (eukaryotic orthologous groups) [28]; Swiss-Prot, a manually annotated and reviewed protein sequence database [29]; KEGG Ortholog database (KO) [30]; and Gene Ontology (GO) [31]. For the nr, nt, KOGs, KEGG, GO, and Swiss-Prot databases, the *E*-value threshold was 1 × 10^−5^. Searches of protein sequences in the public database Pfam (Protein family) [32] were conducted using HMMER 3.1b2 (http://hmmer.org/) [33].

### Prediction of coding regions (CDSs), transcription factors (TFs), and simple sequence repeats (SSRs)

The ANGLE 2.4 software pipeline [34], a long-read implementation of ANGLE, is an error-tolerant method that was used to determine the protein-coding sequences from cDNAs. We used protein sequences from closely related species for ANGEL training and then ran ANGEL prediction using the polished consensus sequences. *Andrias davidianus* TFs were identified using the animalTFDB 2.0 database [35] with default values. SSRs (also known as microsatellite DNA) were identified in the transcriptome using the MIcroSAtellite identification tool (MISA) version:1.0 [36] (http://pgrc.ipk-gatersleben.de/misa/) with default values.

### Prediction of long non-coding RNAs (lncRNAs)

We used Coding-Non-Coding-Index (CNCI) version:2 [37] with default parameters and profiles adjoining nucleotide triplets to classify non-coding transcripts independent of known annotations. We used the NCBI eukaryotic protein database for Coding Potential Calculator (CPC) (Version: cpc-0.9-r2) [38] training; non-coding sequences were discriminated from coding transcripts using an *e*-value of 1*e*-10. We translated each transcript in all three possible frames and used Pfam Scan [33] with default parameters to identify known protein family domains in the Pfam database. Any transcript with a Pfam hit was excluded from subsequent steps. We also filtered out transcripts containing CDSs predicted by ANGLE to obtain non-coding sequences predicted by the three bioinformatics tools.

## Results

### Sequencing of the *A. davidianus* dorsal skin transcriptome using the PacBio sequel system

To obtain the maximum possible number of unigenes and explore the molecular function of *A. davidianus* skin, high-quality RNA was extracted from dorsal skin samples from one animal housed at a farm in Guiding County, Guizhou Province, China. Given the small-fragment sequence bias of the PacBio Sequel System, we constructed two size-fractionated libraries (0.5–4 kb and >4 kb) using the BluePippin Size Selection System to ensure comprehensive coverage of the entire transcriptome. Combined SMRTbell libraries were sequenced on the PacBio Sequel System using four SMRT cells.

### PacBio data processing using SMRTlink

**Figure 1:**
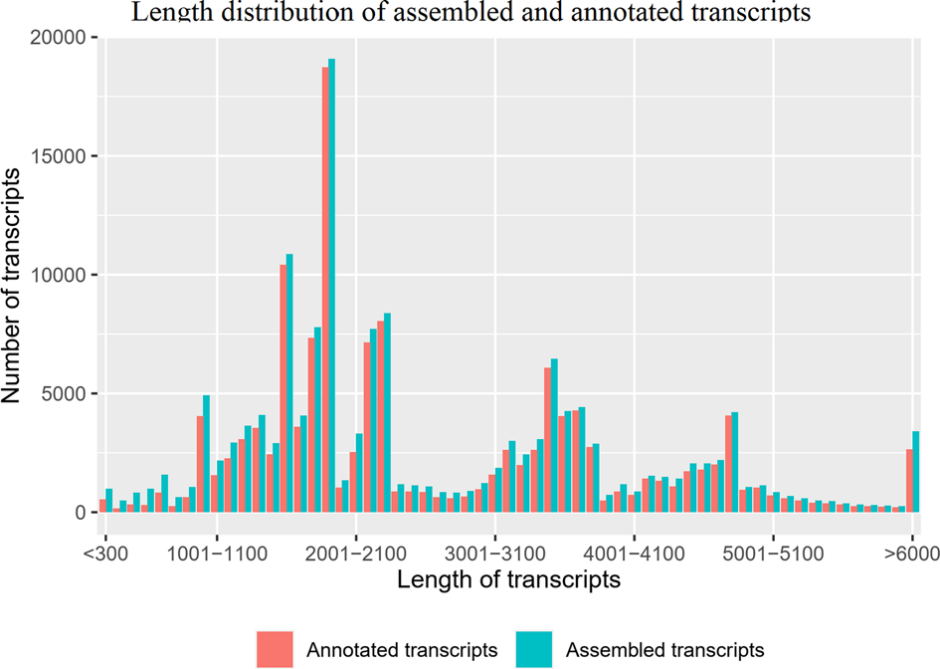
Length distribution of assembled and annotated transcripts

The 0.5–4 kb and >4 kb size-fractionated libraries produced 12.27 and 9.09 GB subreads, respectively; 11.86 GB containing 7,201,899 subreads with an N50 of 1915 bp and 8.79 GB containing 3,693,583 subreads with an N50 of 4589 bp were obtained after removal of SMRTbell adapters and low-quality regions, respectively. To reduce the error rate, all subreads were used to produce CCS reads, which upon further processing generated 219,021 and 98,405 transcripts from the 0.5–4 kb and >4 kb size-fractionated libraries, respectively (Table 1). A total of 153,038 transcripts was generated by combining the two libraries using CD-HIT software [39], including 147,243 (96.2%) full-length non-chimeric (Flnc) transcripts and 5795 (3.8%) non-full-length (non-Fl) transcripts, with unique transcript lengths ranging from 167 to 17,552 bp (Table 1), an N50 of 3432, and an N75 of 1898; the detailed length distribution is shown in Figure 1.

**Table 1:** Summary of transcript assembly information

Library	0.5–4 kb	>4 kb	after clustering	after correction
**Total transcripts**	219,021	98,405	153,038	153,038
**Flnc transcripts**	202,602 (92.5%)	77,445 (78.7%)	147,243 (96.2%)	147,243 (96.2%)
**non-Fl transcripts**	16,419 (7.5%)	20,960 (21.3%)	5795 (3.8%)	5795 (3.8%)
**GC%**	48.20%	53.22%	49.17%	49.17%
**Total length (bp)**	446,609,747	451,427,874	402,093,303	402,093,303
**Mean length (bp)**	2039.1	4587.4	2627.4	2627.4
**N50 (bp)**	2172	4739	3432	3432

### Illumina sequencing and error correction

To obtain accurate long reads, RNA was fragmented to build a cDNA library with an insert size of 350 bp that was sequenced (paired-end, 2 × 150 bp) using an Illumina HiSeq 6000 (Illumina, Inc., San Diego, CA, U.S.A.). We obtained approximately 24.011 Gb of raw data from 160,079,152 reads, of which 23.447 Gb (97.65%) were high-quality; clean data from 156,316,976 reads were used in subsequent analyses. Polished consensus sequences from the PacBio platform were corrected using clean short reads from the Illumina platform by LoRDEC software [25] with 23 k-mers and a solidity abundance threshold of 3 for k-mers.

### Functional annotation of transcripts

**Figure 2:**
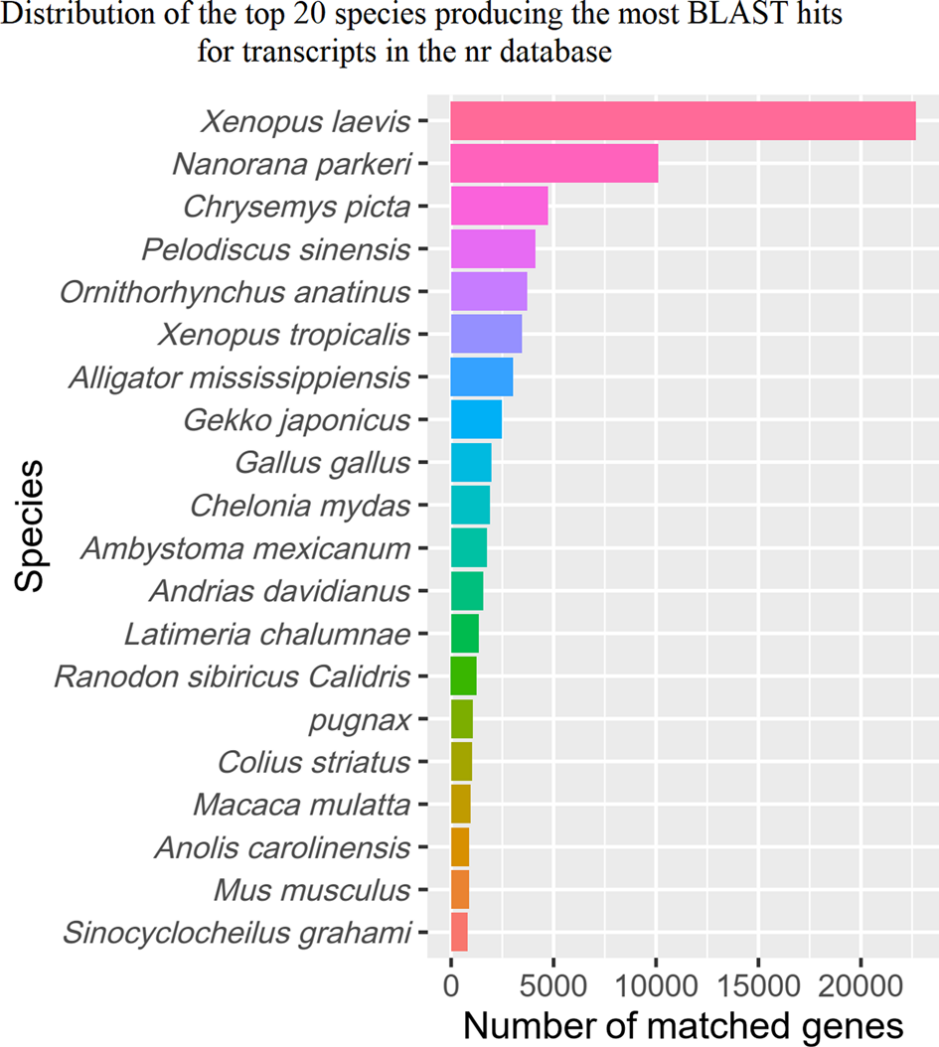
Length distribution of assembled and annotated transcripts

Unique *A. davidianus* transcripts were first annotated using BLAST software [26,40] through homology searches against different protein and nucleotide databases (*E*-value threshold of ≤1*e*-5). A total of 116,825 (76.34%), 109,361 (71.46%), 115,876 (75.72%), 58,732 (38.38%), 68,741 (44.92%), and 94,770 (61.93%) unique transcripts generated significant hits in the nr, Swiss-Prot, KEGG, KOGs, GO, and nt databases, respectively (Supplementary Material S1). Unique *A. davidianus* transcripts were further annotated with HMMER 3.1b2 [33] using data in the Pfam database [32]. A total of 68,741 unique transcripts (44.92%) were assigned using this database. A total of 19,244 transcripts (12.57%) were not identified; 133,794 transcripts (87.43%) were identified in at least one database; and 11,640 transcripts (7.61%) were identified in all databases (Figure 1). The distribution of the top Blastx hits indicated that putative proteins were similar to those of the amphibians *Xenopus laevis* and *Nanorana parkeri* (Figure 2), which suggested that the *A. davidianus* transcriptome was well-assembled.

### Prediction of potential CDSs

**Figure 3:**
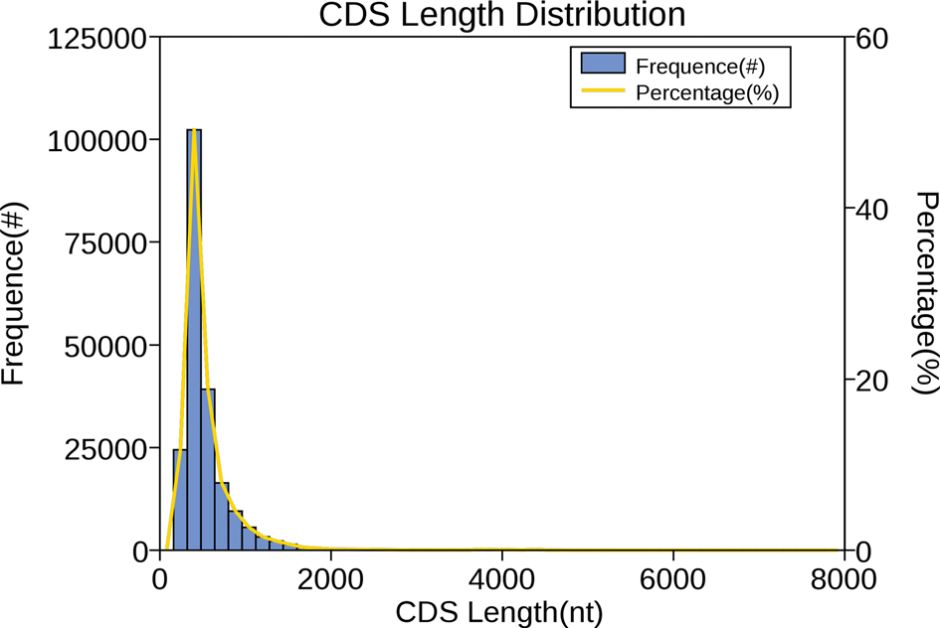
Distribution of CDS length

CDSs were predicted using ANGLE software [34] via an error-tolerant method. A total of 207,627 CDS were obtained from 113,646 unique transcripts (Figure 3). A total of 58,053 transcripts had only one CDS, 33,451 transcripts had two, 13,547 transcripts had three, and all other transcripts had more than three CDS, which likely stemmed from sequencing errors in the PacBio sequencing system. The results of BLAST searches showed that the mean length of CDSs was 539.1 bp, the mean GC content was 53.69%, and the maximum and minimum length of CDSs was 7895 and 294 bp, respectively.

### Identification of possible TFs and candidate lncRNAs

**Figure 4:**
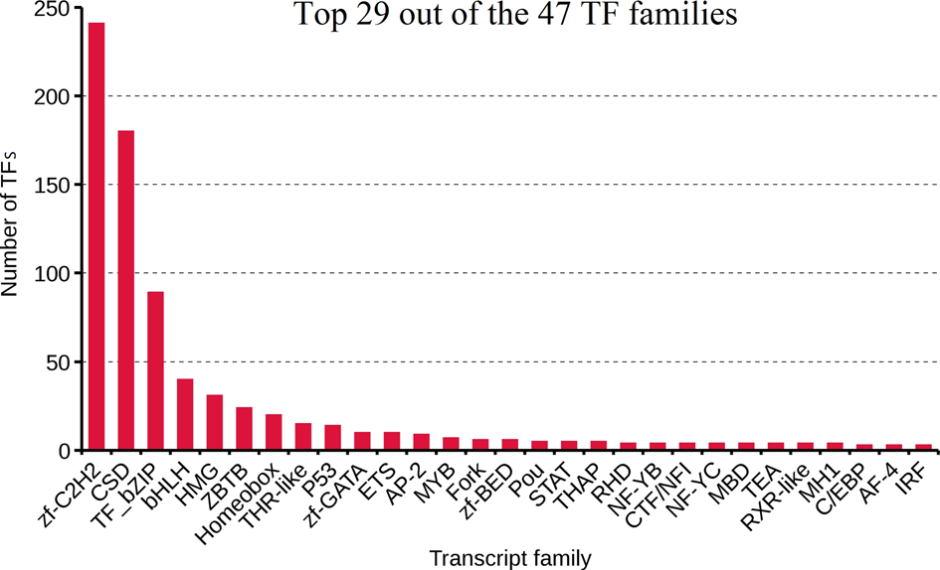
Top 29 out of the 47 TF families

We analyzed TFs and lncRNAs to identify regulatory genes for further studies. We identified and annotated a total of 785 TFs from 47 different families using the animalTFDB 2.0 database [35] (Supplementary Material S2). The top 29 out of 47 TF families are shown in Figure 4. The largest group of TFs was the zf-C2H2 family (241 transcripts; 30.7%), followed by the CSD (180 transcripts; 22.9%), TF_bZIP (89 transcripts; 11.3%), bHLH (40 transcripts; 5.1%), HMG (32 transcripts, 4.1%), ZBTB (24 transcripts, 3.1%), and Homeobox (20 transcripts, 2.5%) families. Together, these seven families represented approximately 80% of the TFs identified among unique *A. davidianus* genes, with the zf-C2H2 and CSD families alone accounting for approximately half.

Non-coding RNAs include tRNAs, small RNAs, microRNAs, rRNAs, and lncRNAs. Our analyses only included lncRNAs containing poly (A) tails identified in the PacBio datasets. Because of the large number of non-coding sequences in the *de novo* dataset, distinguishing non-coding RNA from coding RNA was difficult. We used CNCI [37], CPC [38], and Pfam Scan [33] to predict candidate lncRNAs and then filtered transcripts containing CDSs predicted by ANGLE [34] and transcripts ≤300 bp to identify non-coding sequences generated by the three bioinformatics tools. A total of 27,237 potential lncRNAs were identified with lengths ranging from 300 to 12,850 bp (Supplementary Material S3).

### SSR analysis

**Figure 5:**
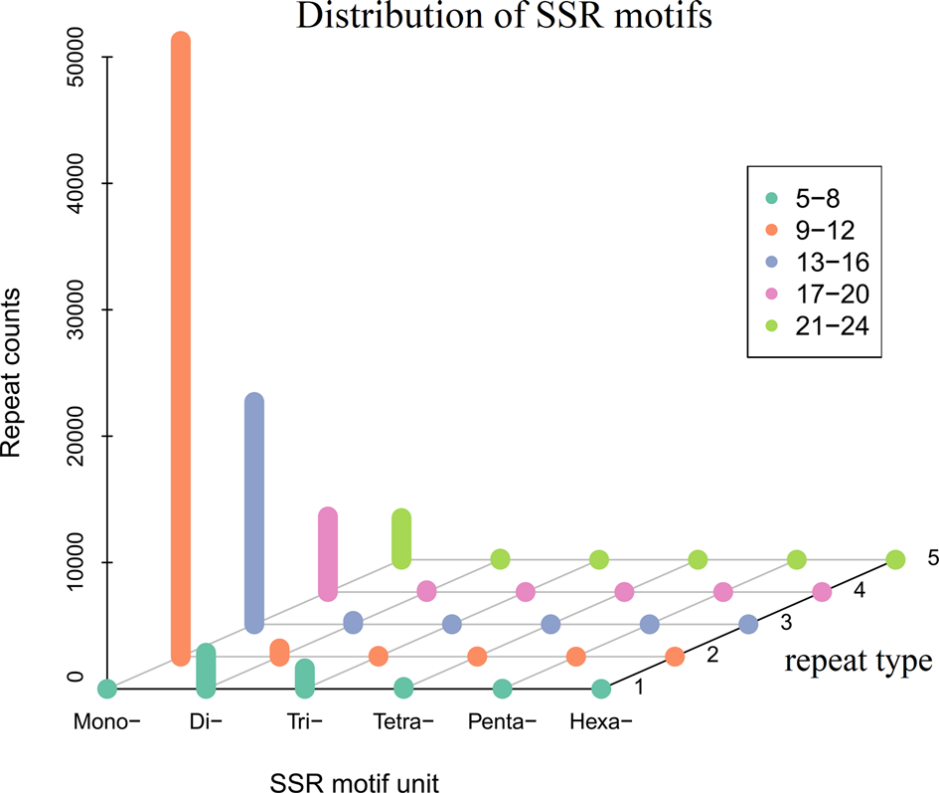
Distribution of SSR motifs

SSRs (microsatellites) are stretches of short tandemly repeated sequences of DNA (1–6 bp) [36]. In this study, we identified 8299 SSR motifs in 49,996 transcripts (32.67%) using MISA software [36] (Figure 5), which comprised 820 simple motifs and 7,479 complex motifs. The longest mono-, di-, tri-, tetra-, penta-, and hexa-nucleotide SSR repeats were A (509), AC (62), TAT (20), ATAG (21), ATGCC (7) and TGGCA (7), and GTTCCA (5), respectively.

### Analysis of eukaryotic orthologous groups (KOGs)

**Figure 6:**
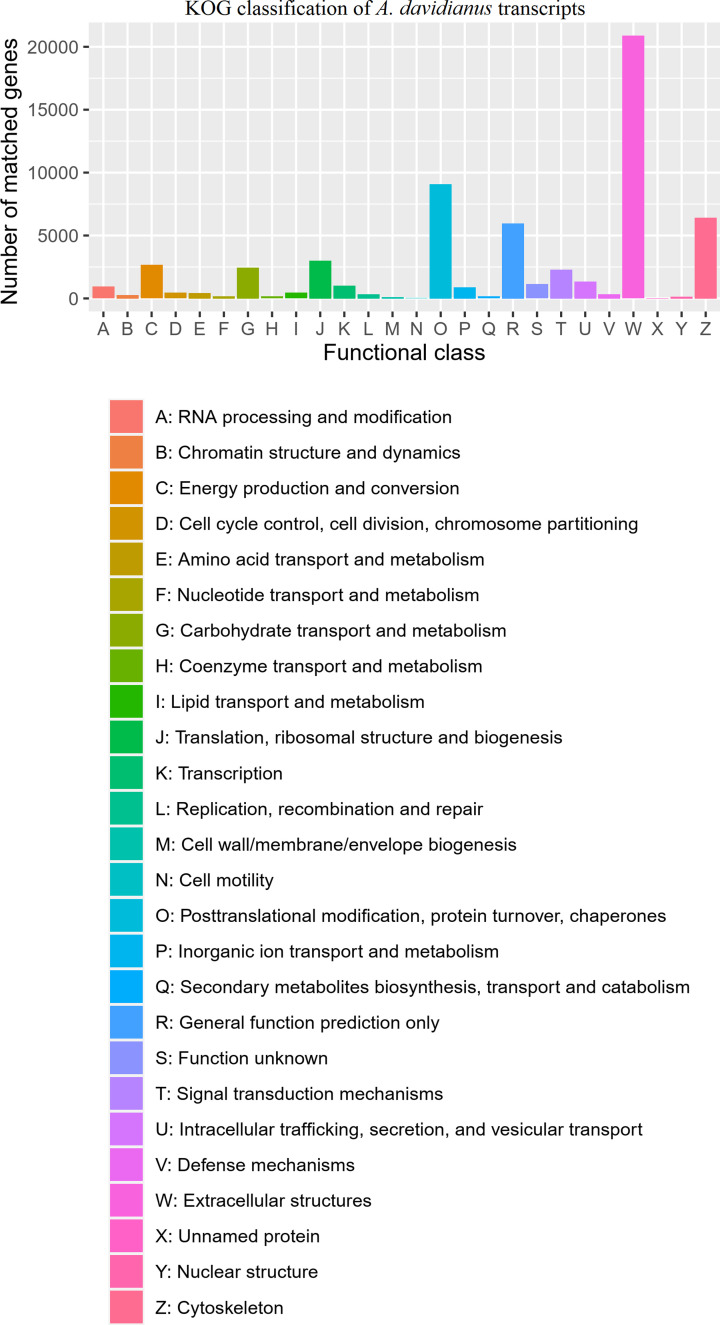
KOG classification of *A. davidianus* transcripts (**A**) RNA processing and modification; (**B**) Chromatin structure and dynamics; (**C**) Energy production and conversion; (**D**) Cell cycle control, cell division, chromosome partitioning; (**E**) Amino acid transport and metabolism; (**F**) Nucleotide transport and metabolism; (**G**) Carbohydrate transport and metabolism; (**H**) Coenzyme transport and metabolism; (**I**) Lipid transport and metabolism; (**J**) Translation, ribosomal structure and biogenesis; (**K**) Transcription; (**L**) Replication, recombination and repair; (**M**) Cell wall/membrane/envelope biogenesis; (**N**) Cell motility; (**O**) Posttranslational modification, protein turnover, chaperones; (**P**) Inorganic ion transport and metabolism; (**Q**) Secondary metabolites biosynthesis, transport and catabolism; (**R**) General function prediction only; (**S**) Function unknown; (**T**) Signal transduction mechanisms; (**U**) Intracellular trafficking, secretion, and vesicular transport; (**V**) Defense mechanisms; (**W**) Extracellular structures; (**X**) Unnamed protein; (**Y**) Nuclear structure; (**Z**) Cytoskeleton.

To identify orthologous protein sets and characterize the functional distribution characteristics of the *A. davidianus* skin transcriptome, a total of 58,732 transcripts were classified into 24 KOG categories (Figure 6). The percentage of annotated unigenes in the “Extracellular structures” (“W” term) category was far higher than that in the other categories (20,868 [13.64%] of the total 153,038 transcripts), and this term was enriched in both size-fractionated libraries (0.5–4 kb and >4 kb). The “R” term category “General function prediction only” containing 5952 transcripts (3.89%) was also enriched in the two libraries. The “O” term category “Posttranslational modification, protein turnover, chaperones” containing 9086 transcripts (5.94%) was primarily enriched in the >4 kb library, and the “Z” term category “Cytoskeleton” containing 6,411 transcripts (4.19%) was mainly enriched in the 0.5–4 kb library.

### GO analysis

**Figure 7:**
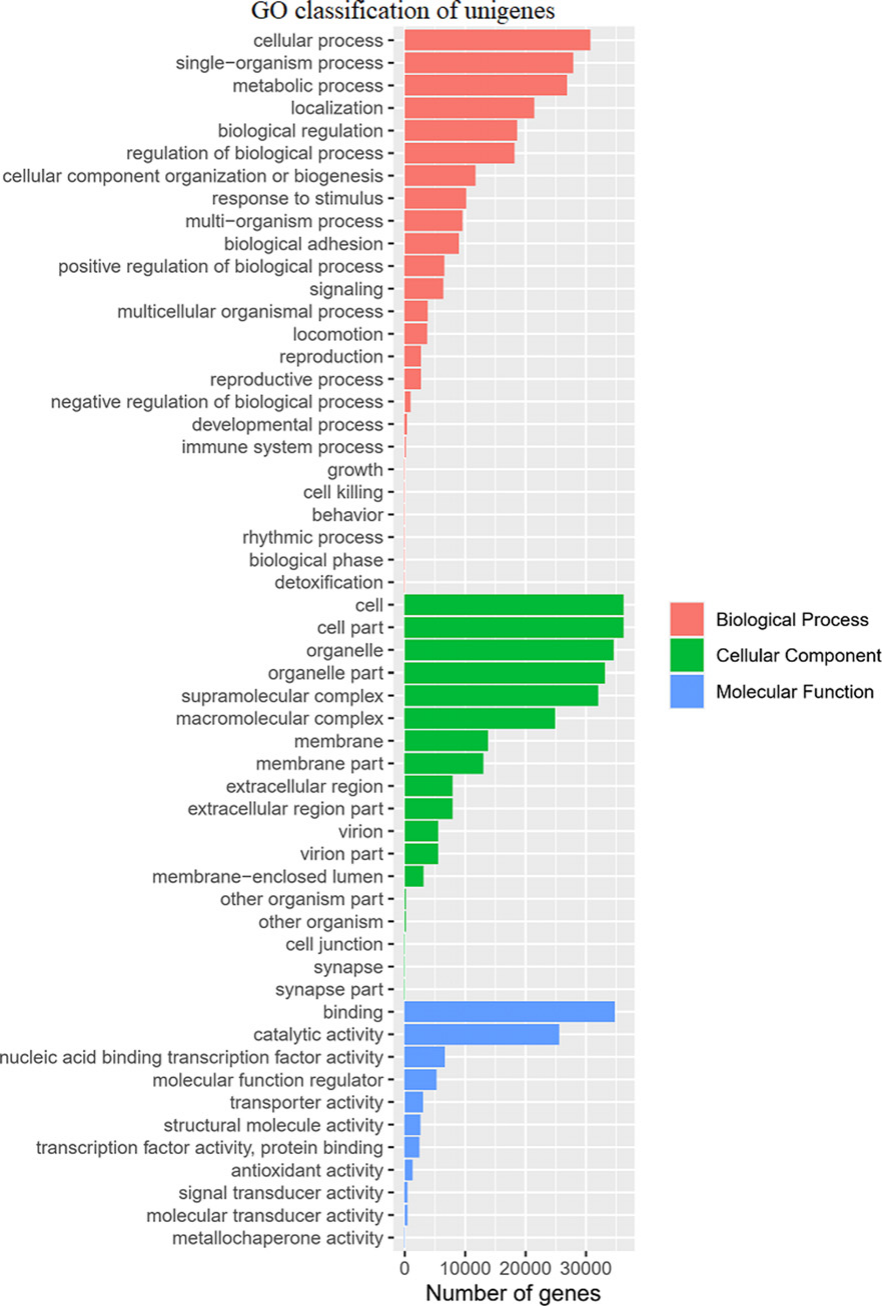
GO classification of unigenes

To identify the functional distribution of unique genes expressed in *A. davidianus* skin, a total of 68,742 transcripts were classified into 1903 GO term categories in the three main classes (GO level 1): biological process, cellular component, and molecular function (Figure 7). The largest numbers of annotated transcripts were in cellular component; among these, the highest proportion of unigenes were enriched in the terms “cell” (14.27%), “cell part” (14.27%), “organelle” (13.61%), “organelle part” (13.05%), and “supramolecular complex” (12.61%). In biological process, transcripts were mainly enriched in the terms “cellular process” (14.56%), “single-organism process” (13.19%), and “metabolic process” (12.71%). In molecular function, the term “binding” (42.23%) was dominant, followed by the term “catalytic activity” (30.05%). Both cellular component and molecular function were enriched in the >4 kb library.

### KO analysis

**Figure 8:**
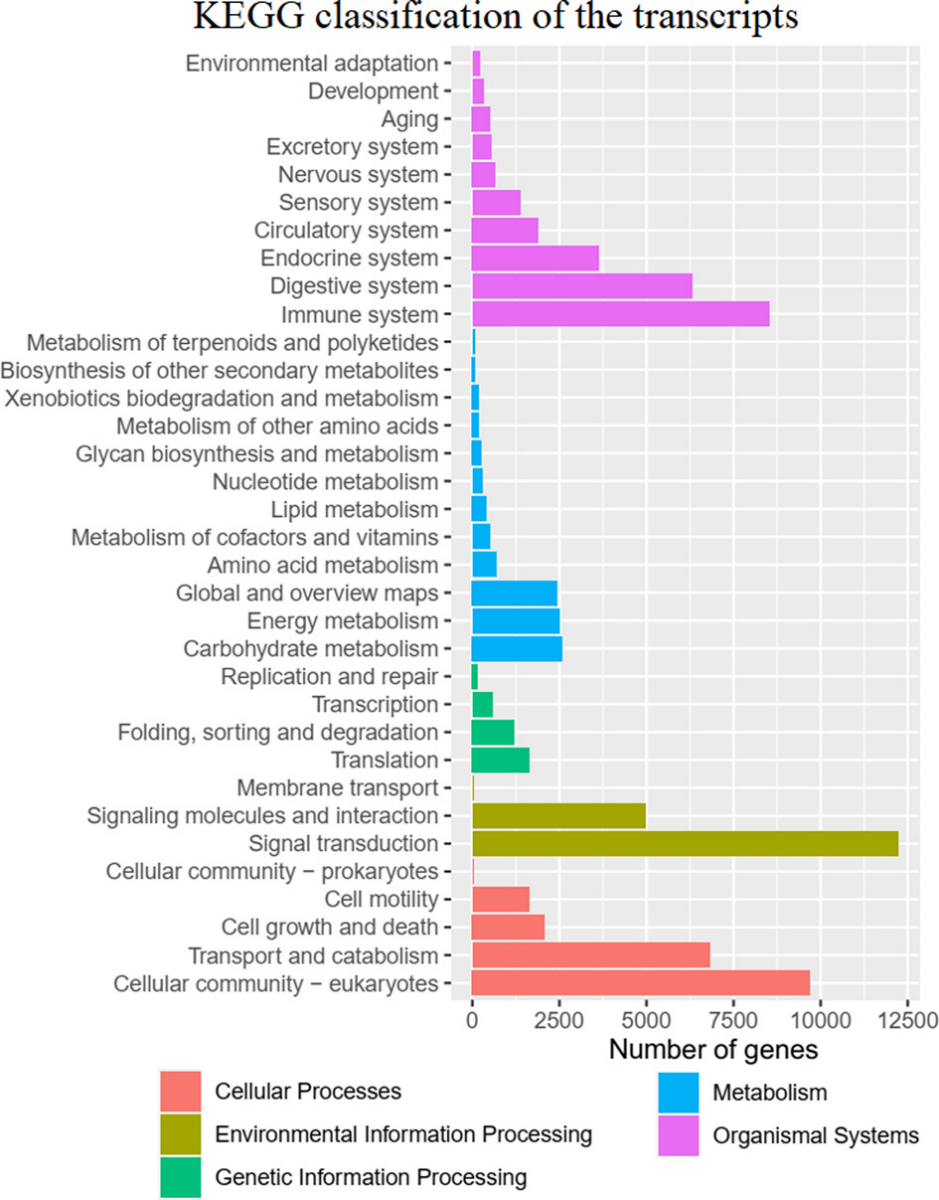
KEGG classification of the transcript isoforms

KO analysis provided additional information on the molecular-level functions of *A. davidianus* skin transcripts, including the metabolic pathways that each transcript isoform contributes to, as one unigene can be assigned to more than one GO term [41]. We investigated 115,876 of the 153,038 transcripts (75.72%), which were enriched in 46 level 2 KEGG pathways (Figure 8); 10,141 transcripts were involved in metabolic pathways. The “signal transduction” pathway was the largest functional pathway (level 2), and a lot of transcript isoforms in both libraries were annotated to “signal transduction” pathways.

## Discussion

We compared hybrid sequencing with NGS in this study. Trinity software [22] assembled all clean reads from the Illumina platform and produced 265,177 transcripts that were hierarchically clustered by Corset [23] to obtain 127,992 unigenes. Differences between hybrid sequencing and NGS were observed (Table 2); specifically, the max length, mean length, N50, N90, and annotated rate were higher for hybrid sequencing than for NGS. According to the annotated results, the gene length distribution was uniform.

**Table 2:** Summary of transcript assembly information

Sequencing technology	Hybrid (This study)	Illumina (This study)	Illumina [9,10]	Illumina [12]	Illumina [11]	Illumina [5]
**Total transcripts**	153,038	127,992	132,912	167,064	93,366	87,297
**GC%**	49.17%	47.96%	49.85%	NA	47.96%	NA
**Max length (bp)**	17,552	15,158	16,067	NA	128,777	NA
**Mean length (bp)**	2627.4	1176	690	647.84	1326	734.80
**N50 (bp)**	3432	2076	1263	956	2409	1216
**N90 (bp)**	1559	476	262	NA	502	NA
**Annotated rate**	87.43%	48.50%	29.85%	33.93%	44.85%	40.06%

Annotated rate: Percentage of unigenes annotated in each database, including nr, Swiss-Prot, KEGG, KOGs, GO, and nt databases. NA: not available.

To ensure the robustness of the hybrid-sequenced transcripts, Illumina short reads were mapped to transcripts using Bowtie2 version 2.2.5 [24] with a mismatch of 0 (Table 3). SRX4453287 (this study) was from dorsal skin tissue; SRR5344016 was from skin tissue [12]; SRR4449143 was from dorsal skin tissue [11]; SRR4449153 was from lateral skin tissue [11]; SRR4449153 was from abdominal skin tissue [11]; and SRR1609131 was from skin tissue [5]. Transcripts were hybrid assembled, and unigenes were assembled using Illumina short reads. CGS_All_Unigene_filter.fa was downloaded from Xiaofang Geng et al. [11] (http://gigadb.org/dataset/100277), which was assembled from more than 20 tissues [11]. The mapping rate of hybrid-assembled transcripts was higher for dorsal skin tissue than for other tissues.

**Table 3:** Mapping rate of Illumina short reads

Transcripts	SRX4453287 (This study)	SRR5344016 [12]	SRR4449143 [11]	SRR4449153 [11]	SRR4449117 [11]	SRR1609131 [5]
**Transcripts by hybrid sequencing (This study)**	80.32%	81.4%	81.41%	65.4%	71.72%	80.73%
**Unigenes by Illumina (This study)**	78.07%	77.85%	79.6%	68.16%	72.36%	81.64%
**CGS_All_ Unigene_filter.fa [11]**	77.39%	80.43%	82.64%	79.58%	81.18%	81.50%

SRX4453287 was from dorsal skin tissue; SRR5344016 was from skin tissue; SRR4449143 was from dorsal skin tissue; SRR4449153 was from lateral skin tissue; SRR4449153 was from abdominal skin tissue; and SRR1609131 was from skin tissue. Transcripts were hybrid assembled, and unigenes were assembled using Illumina short reads. CGS_All_Unigene_filter.fa was downloaded from Xiaofang Geng et al. [11] (http://gigadb.org/dataset/100277), which was assembled from more than 20 tissues [11].

According to the assembly results of the two libraries, the percentage of full-length unigenes was higher in the 0.5–4 kb library than in the >4 kb library, likely because of the sequence length bias of the PacBio sequencing system. The GO category biological process was lacking in the > 4 kb library, which differed from the 0.5–4 kb library. According to KO annotation, the term “immune system” was more enriched among transcripts in the >4 kb library than in the 0.5–4 kb library. These annotated functional genes will aid future cloning and analysis of the full-length sequences of these transcripts.

Hybrid sequencing analysis recovered more full-length transcripts, had higher N50 contig lengths, and had more CDSs and predicted unique genes compared with previous studies based on NGS. Exploration of the molecular basis of amphibian skin function and adaptation requires understanding the evolutionary processes affecting amphibians (Li et al., 2016). Sequencing of the skin transcriptome of *A. davidianus* generated 153,038 transcripts from two size-fractionated libraries (0.5–4 kb and >4 kb). This large dataset has the potential to aid our understanding of the molecular-level function of amphibian skin.

Combining the proteome and metabolome with this reference dataset could facilitate the identification of functional proteins and their structures, such as immune proteins and colloid proteins; single-cell sequencing; analyses of the function of giant salamander skin; analysis of the transcriptome associated with biological stress; the construction of a transcriptional regulation network; and the protection of giant salamanders.

## Supplementary Material

Supplementary Materials S1-S3

## Data Availability

The raw data can be obtained from the Sequence Read Archive at NCBI under the accession numbers SRX3734340, SRX3734347, and SRX4453287, which are a part of BioProject PRJNA433898 (SRP133300).

## Abbreviations

CD: coding region
lncRNA: long non-coding RNA
SSR: simple sequence repeat
TF: transcription factor
